# Ischemic Stroke among the Symptoms Caused by the COVID-19 Infection

**DOI:** 10.3390/jcm9092688

**Published:** 2020-08-19

**Authors:** Rafal Szelenberger, Joanna Saluk-Bijak, Michal Bijak

**Affiliations:** 1 Department of General Biochemistry, Faculty of Biology and Environmental Protection, University of Lodz, Pomorska 141/143, 90-236 Lodz, Poland; rafal.szelenberger@unilodz.eu (R.S.); joanna.saluk@biol.uni.lodz.pl (J.S.-B.); 2 Biohazard Prevention Centre, Faculty of Biology and Environmental Protection, University of Lodz, Pomorska 141/143, 90-236 Lodz, Poland

**Keywords:** COVID-19, SARS-CoV-2, coagulopathy, thrombosis, stroke

## Abstract

The 2019 global pandemic of coronavirus disease 2019 (COVID-19) caused by the severe acute respiratory syndrome coronavirus 2 (SARS-CoV-2) has been declared a public health emergency of international concern by the World Health Organization (WHO). The WHO recognized the spread of COVID-19 as a pandemic on 11 March 2020. Based on statistics from 10 August 2020, more than 20.2 million cases of COVID-19 have been reported resulting in more than 738,000 deaths. This completely new coronavirus has spread worldwide in a short period, causing economic crises and healthcare system failures worldwide. Initially, it was thought that the main health threat was associated with respiratory system failures, but since then, SARS-CoV-2 has been linked to a broad spectrum of symptoms indicating neurological manifestations, including ischemic stroke. Current knowledge about SARS-CoV-2 and its complications is very limited because of its rapidly evolving character. However, further research is undoubtedly necessary to understand the causes of neurological abnormalities, including acute cerebrovascular disease. The viral infection is inextricably associated with the activation of the immune system and the release of pro-inflammatory factors, that can stimulate the host organism to defend itself. However, the body’s immune response is a double-edged sword that on one hand, destroys the virus but also disrupts the homeostasis leading to serious complications, including thrombosis. Numerous studies have linked coagulopathies with COVID-19, however, there is great uncertainty regarding it functions on the molecular level. In this review, a detailed insight into the biological processes associated with ischemic stroke in COVID-19 patients and suggest a possible explanation for this phenomenon is provided.

## 1. Introduction

Coronavirus disease 2019 (COVID-19) is a new infectious disease caused by the newly identified severe acute respiratory syndrome coronavirus 2 (SARS-CoV-2), which was classified by the World Health Organization (WHO) as a pandemic on 11 March 2020. Belonging to the *Orthocoronavirinae* subfamily in the *Coronaviridae* family, SARS-CoV-2 is the seventh member of all coronaviruses with the ability to infect humans [1]. As regards its origin, there are few theories, the most probable one being that SARS-CoV-2 has a natural, zoonotic origin. It is closely related to bat coronaviruses, pangolin coronaviruses and SARS-CoV. The first diagnostic reports of an unusual respiratory disease appeared in December 2019 in the city of Wuhan (Hubei province), China and were linked to a cluster of wet markets processing bat meat and their guano [2]. However, new reports from China suggest that a 55-year-old person from Hubei may have been the first person infected on 17 November 2019. Isolation and genome sequencing of the new virus led to the discovery of a new pathogen that primarily caused “pneumonia of an unknown etiology” [1]. However, current knowledge about this virus is very limited and is mostly derived from previous coronaviruses. Longitudinal serological and immunological studies are necessary to assess the efficiency of an immune response to SARS-CoV-2 [3].

Initially, SARS-CoV-2 was thought to cause fever, dyspnea, cough and fatigue via infection of the host’s respiratory system [2]. However, the ongoing scientific effort in order to profiling of COVID-19 patients revealed that patients exhibit a broader range of atypical symptoms affecting the severity and disease progression, including headache, nasal congestion, diarrhea, loss of taste or smell, rash and conjunctivitis [4]. Furthermore, there is the onset of a wide range of symptoms, the presence of comorbidities and response to existing therapies failure, which may result in mild pneumonia that quickly develops into the acute respiratory syndrome sepsis, and even to multi-organ dysfunction within a short period of time [5].

The vast majority of SARS-CoV-2 infections are asymptomatic at the time of testing. However, most of infected people developed symptoms later, which enhance the virus transmission. Furthermore, the presence of flu-like symptoms with a prolonged viral incubation period may result in wrong diagnosis or the disease not being detected at all. Not isolated and infected individuals are a vector for the rapid spread and advanced migration of SARS-CoV-2. An estimation of the basic reproduction number (R0) for COVID-19 in January showed that it may be about 5.7 (with a 95% confidence interval of 3.8 to 8.9). The low mortality threshold facilitates a host-to-host transfer, increasing the number of cases exponentially [6].

According to the 10 August 2020 WHO data, more than 20.2 million cases of COVID-19 have been reported resulting in more than 738,000 deaths. This number will continue to grow unless an effective treatment or vaccine is developed.

The appearing threat associated with COVID-19 pandemic is related to the virus ability to induce microvascular, venous and arterial thrombosis, thus exacerbating the functionality of organs. Many clinical studies have shown an association between SARS-CoV-2 infection and hypercoagulability diagnosed on the basis of abnormal coagulation parameters, including activated partial thromboplastin time, prothrombin time, fibrinogen, D-dimer and C-reactive protein level. Furthermore, studies showed that ischemic events, including venous thromboembolism, were present in 25–49% of patients with severe viral infection. Statistics proved that patients with thrombotic complications have 5-fold augmented mortality. What is more, autopsy series on COVID-19 non-survivors found not only macrovascular complications, but also microvascular thrombosis. Small thrombi were found in over 80% samples of pulmonary vasculature. Several groups reported also augmented rates of ischemic stroke in COVID-19 patients admitted to hospital [7].

All those evidence indicate that SARS-CoV-2 may contribute to a number of vascular disorders, indicating the necessity for detailed patients diagnoses in order to avoid further complications that significantly reduce life quality. In this review, the potential mechanism and the effect of the SARS-CoV-2 viral infection on the development of ischemic stroke in COVID-19 patients were carefully studied.

## 2. Thrombosis and COVID-19

Thrombosis is a pathological process associated with the blood clots formation in circulatory system. Thrombosis may occur within the venous and arterial system and contribute to various medical complications, including stroke, myocardial infarction or pulmonary embolism [8]. As mentioned above, many studies confirm the presence of thrombosis in patients diagnosed with COVID-19. Although studies do not implicate SARS-CoV-2 to have procoagulant effect itself, scientists more likely assess COVID-19 coagulopathy with profound inflammatory response [9].

Spreading the viral infection can contribute to the formation of many inflammatory foci in the human body in various places. The proliferation of the virus in the lungs causes diffuse interstitial and alveolar inflammatory exudation, which leads to edema and gas exchange disorders, resulting in hypoxia in the central nervous system (CNS). Thus increasing oxygen-free metabolism in the brain cells mitochondria [10,11]. What is more, rapidly progressing inflammation, activation of the coagulation system and an imbalance between pro- and anti-coagulant properties may lead to the formation of disseminated intravascular coagulation (DIC) syndrome. Moreover, a systematic disorder characterized by a widespread activation of the hemostatic system leading to excessive blood clot formation in small vessels with simultaneous, massive consumption of blood platelets and coagulation factors, resulting in hemorrhagic complications are observed [12,13]. The presence of DIC was confirmed by the Tang et al. study, in which most non-survivor COVID-19 patients’ (71.4%) blood tests showed prolonged prothrombin time and an increased D-dimer levels, which indicated the state after activation of the plasma coagulation system [14]. Data from many studies showed a significant decrease in the platelet count, increased fibrinogen and D-dimer levels and prolonged prothrombin time, which was associated with severe COVID-19 infections. Thus indicating excessive activity of the coagulation system and the risk of DIC development [10,15,16,17]. Ranucci et al., besides the augmented level of fibrinogen and D-dimer levels, also presented a significant increase of IL6 and antithrombin levels, prolonged coagulation indicator-activated partial thromboplastin time (APTT) and elevated parameters of blood viscoelasticity [18]. Coagulation changes were also proven by Magro et al. in lung histopathological analysis and skin biopsies, which showed generalized microvascular thrombotic disorder [19]. Furthermore, in a study conducted by Carsana et al., a pulmonary autopsy showed that small arterial vessel fibrin thrombus was observed in 86.8% of examined, non-survived patients [20].

## 3. Ischemic Stroke

### 3.1. Epidemiology of Stroke

Stroke is a medical condition caused by a deficit of blood flow in the brain causing neurological dysfunctions [21]. Global epidemiologic reports ranked stroke as the second death cause globally, with a mortality rate of approximately 5.5 million per year. Stroke survivors are at high risk of chronic disability leading to loss of their independence, work capacity, employment and material resources [22]. A sudden loss of neurological function is caused by infarction or cerebral vessels hemorrhage, the spinal cord or retina. Clinically, patients mostly experienced unilateral weakness, ataxia, altered speech, numbness and/or visual loss. However, atypical symptoms like amnesia, dysphagia, dysarthria, anosognosia, headache and confusion may occur simultaneously [23].

The term “stroke” is not commonly used in clinical practice, because of its various etiology. The most common and generally diagnosed subtype of stroke is ischemic stroke, which constitutes 88% of all diagnosed cases. This subtype of stroke is caused by a partial or complete blockage of blood flow in the brain, which results in cerebral ischemia. A reduction in blood circulation to 16 mL/100 g of the brain tissue per minute may cause irreversible tissue damage within one hour. Moreover, full occlusion and the absence of blood flow leads to the death of brain cells within 4 to 10 minutes [21]. Most commonly, ischemia is caused by local vessel injury as an effect of atherosclerosis. The formation of plaque in the vessel lumen begins with damaged endothelium, ongoing inflammation and activation of the coagulation system. Along with the increased severity of pathological processes, plaque forms become thicker and fibrous. In the final step, a clot that forms may partially or completely limit the blood flow in the vessels, or break free, forming an embolus, which is able to travel through vessels and block the blood flow further on [21].

A cerebral hemorrhage is the next subtype of stroke, caused by the rupture of a cerebral vessel, resulting in extravasation of blood within the brain [21]. Generally, hemorrhagic stroke is a complication of hypertension, cerebral amyloid angiopathy, anticoagulation therapy and/or vascular structural lesions [23]. Symptoms may vary between patients, depending on the anatomical site of the hemorrhage [21].

The major risk factors for the stroke development are: modifiable and include hypertension, atrial fibrillation and atrial cardiopathy, dyslipidemia, obesity, lack of physical activity, diet, untreated co-morbidities and inflammation, alcohol consumption and smoking. Mostly, they contribute to the elevation of blood pressure and the progression of atherosclerosis. Health improvement associated with the elimination of behavioral and medical risk factors can significantly reduce the risk of stroke. However, non-modifiable risk factors including age, sex, genetics and ethnicity can also increase the chance of stroke development [24].

Identification of a stroke syndrome is usually easy to recognize because of visible neurologic deficits. However, symptoms differ among various regions of the brain and types of stroke. Therefore, neuroimaging is a gold standard method for all stroke diagnostics. The vast majority of strokes may be recognized using FAST acronym, which means Facial droop, Arm droop, Speech disturbances and Time. Computed tomography (CT) is the first examination that can with almost 100% certainty confirm stroke and in over 95% accuracy assess the type of stroke. However, small-volume ischemia may not be detected in CT because of insufficient resolution. For higher resolution, magnetic resonance imagining (MRI) is recommended. For all acute stroke syndromes, CT angiography is recommended due to the identification of ischemic area. The determination of occlusion and evaluation of extracranial vertebral and carotid, aortic arch and proximal great vessels is necessary for further management. Although patients with acute coronary syndromes have helpful diagnostic biomarkers (i.e., serum troponin, electrocardiography), for stroke patients those tests are not available [25]. Despite the available clinical studies evaluating the potential role of hemostatis biomarkers (i.e., von Willebrand factor (vWF), P-selectin, fibrinogen, thrombomodulin, tissue factor, d-dimer, etc.) in ischemic stroke patients, the value of studied biomarkers is still unproven and requires further investigation [26].

### 3.2. Molecular Pathophysiology of Ischemic Stroke

**Figure 1:**
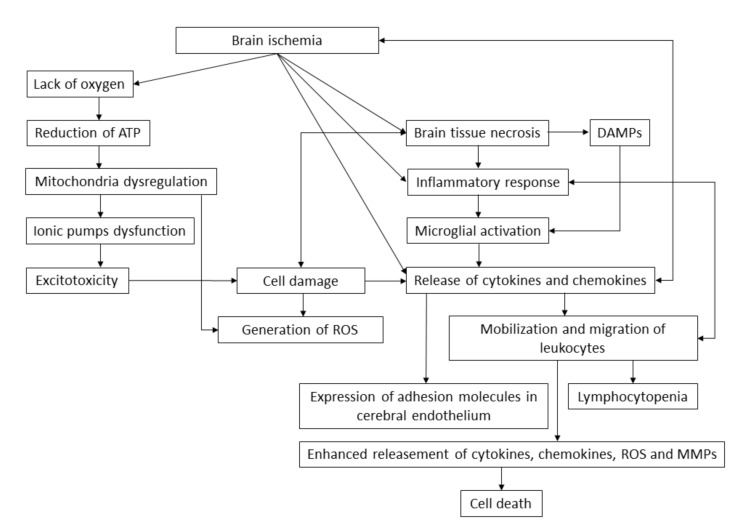
The brain ischemia pathway. Brain ischemia causes shortages in the oxygen supply, brain tissue necrosis and release of cytokines and chemokines that cause an inflammatory response. Lack of oxygen causes the dysregulation of mitochondria and induces the anaerobic production of adenosine triphosphate (ATP), which generates the reactive oxygen species (ROS). Disorders in the concentration of ions cause excitotoxicity, which results in cell damage and brain tissue necrosis. Necrotic cells release damaged-associated molecular patterns (DAMPs), which induce the activation of microglia, resulting in a massive release of cytokines and chemokines. Pro-inflammatory factors mobilize leukocytes to migrate into the infarct zone enhancing the release of inflammatory response molecules. Cerebral endothelium is stimulated to express the adhesion molecules on its surface and accumulate the cells, narrowing the vessel lumen and elevating the formation of atherosclerotic plaque. The ongoing mobilization of leukocytes results in the immunodeficiency caused by lymphocytopenia, thus increasing the risk of infection, which complicates the stroke by increasing the activation of the immune system and its interaction with endothelial and neural cells.

Ischemic stroke is a dynamic process that persists for more than 24 h. An ischemic cascade is activated rapidly after lack of blood flow in the brain, resulting in an ionic imbalance, excitotoxicity, blood–brain barrier dysfunction, generation of nitrosative and oxidative stress and inflammation (Figure 1). Shortages in glucose and oxygen delivery, caused by the ischemic event, force the human body to use alternative biochemical pathways and substrates like glycogen, fatty acids or lactate. However, lack of oxygen leads to the reduction of adenosine triphosphate (ATP) (inducing glycolytic metabolism), accumulation of lactate and protons and diminishment in intracellular pH. Dysfunction in the activity of the electron transport chain in mitochondria causes a further reduction in ATP concentration and disturbances in the functioning of ionic pumps. A loss of potassium ion concentration and an increase in sodium, chloride and calcium ion concentration leads to the depolarization of the cell membrane of astrocytes and neurons and to the secretion of neurotransmitters causing excitotoxicity [27]. During the excitotoxicity process, neuronal cells are exposed to a high amount of glutamate. The augmented concentration of glutamate may occur after neuronal depolarization, which is excessively released after neuronal depolarization. Increased exposition of brain tissue to glutamate induces neuronal death, mitochondria failure and apoptosis. An influx of calcium ions causes degeneration of organelles and disrupts the integrity of cellular membrane [28]. Removal of excess calcium ions is possible through ATP-dependent mitochondria activity. However, this involves the production of reactive oxygen species (ROS), thus inducing the peroxidation of lipids, activation of proteases, disruption of cell membrane integrity, dysfunction of mitochondria, stimulation of microglia and production of cytotoxic factors. During shortages of oxygen and glucose, mitochondria switches to anaerobic ATP production, resulting in the formation of lactic acid and hydrogen ions, which provide a substrate for the conversion of superoxide anion into hydrogen peroxide or hydroxyl radical. Along with nitrogen oxides, oxidative and nitrosative stress increase, thus enhancing brain tissue damage. Ongoing ischemia and associated pathological processes cause necrotic cell death [27], which induces the release of damaged-associated molecular patterns (DAMPs), endogenous biomolecules responsible for the activation of the innate immune system from dead cells [29]. Ischemic stroke also triggers the inflammation of the brain tissue as a result of oxidative and nitrosative stress and the formation of free radicals, hypoxia or necrotic cell death [27]. The inflammatory response to ischemia causes the rapid activation of microglial cells, which induce the infiltration of circulating inflammatory cells. Ischemic cell damage generates and releases pro-inflammatory mediators and ROS, thus promoting transendothelial migration of circulating leukocytes and inducing the expression of adhesion molecules in endothelial brain cells. Within hours and days, mobilized leukocytes release chemokines, cytokines and ROS, which enhance the inflammatory response in brain tissue [30]. Circulating monocytes activated by cytokine storm and chemotactic factors roll from the central axis to the peripheral marginal bloodstream and bind with the endothelium surface. The rapidly repeating and overlapping processes of cytokine releasement, monocyte migration and its binding with endothelium cause excessive cell accumulation. Trapped monocytes undergo a transformation process into macrophages, which intensively internalize and accumulate lipids, thus transforming into foam cells [31]. Oxidized low-density lipoproteins inhibit a tethered macrophages chemotaxis, thus preventing them from leaving the endothelium and amplifying the accumulation [32]. The leukocytes sequential migration causes lymphocytopenia, which contributes to the increased risk of infection via immunodepression [28]. The ongoing pathological state results in the expression of pro-inflammatory genes and the augmented production of pro-inflammatory factors via the NF-κB pathway. Intra- and extracellular signaling pathways trigger the interaction among brain tissue, endothelial cells, immune cells and hemostatic cells, thus stimulating the release of cytotoxic molecules like matrix metalloproteinases (MMPs), which initially, causes the disruption of blood–brain barrier (BBB) permeability, nitric oxide, which constitute an independent source of reactive nitrogen species and DAMPs, which enhance the cells mobilization and migration. Disruption of BBB permits the infiltration of leukocytes, neurotoxic substances, cytokines, chemokines and pathogens to enter the brain tissue, exaggerating the infarct zone and resulting in the microvascular occlusion [27,33].

Neuronal damage caused by brain injury may be monitored by some brain markers including S100B protein and neuron-specific enolase (NSE). S100B belongs to the Ca^2+^ binding protein family and is responsible for intracellular level of Ca^2+^ ions regulation. The concentration of S100B in cerebrospinal fluid and plasma is correlated with brain damage and disease severity. Serum S100B levels are 40-fold decreased in comparison to cerebrospinal fluid level, however, serum protein is significantly easier and less invasive to collect and measure. Several studies concluded that serum S100B level shows strong correlation with the volume of infarct and the size of neurological deficit [34]. NSE is an isoenzyme of the enolase found in neuron’s cytoplasm and is considered as neuronal damage biomarker. NSE is present in peripheral blood serum in negligible concentration and its level rise during cell death. The study conducted by Bharosay et al. has shown that NSE serum level increases significantly due to cerebrovascular stroke (*p* < 0.001) and is correlated with score and disability degree [35]. Both neuronal damage biomarkers have a potential to be use in the determination of the reason of brain damage (injury caused by SARS-CoV-2, or injury caused by stroke). However, there are currently no studies that describe this association.

The contribution of viral infection in atherogenesis has been discussed for many years. Studies showed that viral infection can be associated with endothelial dysfunction, the progression of atherosclerosis and future cardiovascular mortality. Pathogens residing in the vascular wall induce the response of the immune system and the endothelium dysfunction, promoting the inhibition of vasodilatation, elevating the expression of pro-inflammatory factors and reactive oxygen species (ROS), as well as contributing to the rupturing of plaque caused by MMP activity. Unfavorable features of an ongoing pathological state of viral infection devastate the host organism and may contribute to severe complications of the initial pneumonia [32].

## 4. The Association between COVID-19 and Ischemic Stroke

### 4.1. The Clinical Characteristics of COVID-19 Patients

The formation of blood clots in the cerebral vessel as a complication of SARS-CoV-2 infection, has been reported in a significant number of research articles. In a study conducted by Mao et al., of the 214 patients diagnosed with COVID-19 who enrolled for their study, 78 had neurological disorders categorized into three categories: CNS, which included headache, dizziness, impaired consciousness, ischemic stroke and cerebral hemorrhage; skeletal muscular injury defined as pain muscle or augmented level of serum creatine kinase (higher than 200 U/I); and peripheral nervous system (PNS), which included smell, taste or vision impairment, and/or nerve pain. CNS symptoms were the most relevant among all the neurological manifestations in patients. Of 5 patients with diagnosed ischemic stroke, only one survived. The authors showed that patients with CNS symptoms had lower platelet counts, lower lymphocyte levels and augmented blood urea nitrogen levels compared to patients without CNS symptoms. What is more, patients with severe infections had augmented D-dimer levels [10]. Similar results were conducted by Beyrouti et al., where the clinical characteristics of six patients were presented. The first patient, a 64-year-old man diagnosed with COVID-19 and exhibiting symptoms like cough, fever, breathlessness, myalgia and poor appetite was admitted to the intensive care unit due to respiratory failure. During hospitalization, the patient developed mild left upper limb weakness and incoordination. Magnetic resonance imaging (MRI) showed acute left posterior inferior cerebellar artery territory infarct with petechial hemorrhage and intradural left vertebral artery occlusion. Moreover, the patient had markedly elevated D-dimer levels (>80,000 µg/L). The patient’s deteriorating health revealed a bilateral pulmonary embolism and acute bilateral incoordination, high homonymous hemianopia and extensive acute posterior cerebral artery territory infarction diagnosed with MRI. The second patient was a 53-year-old woman with valvular atrial fibrillation and confirmed COVID-19 with cough, dyspnea, acute confusion, incoordination and drowsiness. A computed tomography (CT) scan showed acute large left cerebellar and right parieto-occipital infarcts. At the time of the stroke, there was an onset of symptoms: the patient had augmented D-dimer levels (7750 µg/L) and a prolonged prothrombin time with an international normalized ratio (INR) of 3.6. Cardiorespiratory deterioration and disease severity contributed to the patient’s decease. The third patient, an 85-year-old man diagnosed with COVID-19 and risk factors like hypertension, atrial fibrillation and ischemic heart disease, developed a left posterior cerebral artery occlusion and infarction confirmed with a CT scan. The D-dimer levels were also highly increased (16,100 µg/L). The fourth patient, a 61-year-old man admitted to the hospital with hypertension, a high body mass index and previous stroke history at the time of the medical interview, had acute right striatal infarct detected by a brain MRI, and markedly elevated D-dimer levels (27,190 µg/L). During hospitalization, the patient developed respiratory symptoms with a pulmonary embolus confirmed with CT angiogram and was diagnosed with COVID-19. The fifth patient, an 83-year-old man diagnosed with COVID-19, diabetes, hypertension, smoking and alcohol consumption and ischemic heart disease, developed a thrombotic occlusion of proximal M2 branch of the right middle cerebral artery and infarct in the right insula. Similarly to all patients, the D-dimer levels were augmented (19,450 µg/L). The final and sixth patient, a 70-year-old man with common COVID-19 symptoms, was admitted to the hospital with dysphasia and right hemiparesis. An MRI brain test confirmed bilateral P2 segment stenosis, thrombus in the basilar artery and multiple acute infarcts in the left pons, right thalamus, right cerebellar hemisphere and right occipital lobe. The D-dimer levels were 1080 µg/L and measured after intravenous thrombolysis. Based on their observations, the authors suggest that ischemic stroke is a complication of COVID-19, and may have distinct characteristics. However, the mechanisms of this disorder are not yet understood [16].

Oxley et al. published a case report study, in which five patients younger than 50 years of age, diagnosed with COVID-19, developed a large-vessel stroke. The first and second patients were a 33-year-old female and 37-year-old man, respectively, displayed no risk factors for stroke in their medical records. The female patient had mild COVID-19 symptoms like cough, headache and chills. Medical tests showed a partial infarction of the right middle cerebral artery with a partially occlusive thrombus in the right carotid artery at the cervical bifurcation, hemiplegia on the left side, dysarthria, sensory deficit, homonymous hemianopia and facial droop. The male patient, recently exposed to a SARS-CoV-2 infected family member, showed no symptoms of COVID-19. However, medical tests confirmed ischemia in a left middle cerebral artery, and stroke symptoms such as sensory deficit, dysarthria, hemiplegia on the right side, reduced consciousness and dysphasia. Other patients, a 39-year-old man, a 44-year-old man and a 49-year-old man were diagnosed with ischemia in a right posterior cerebral artery, left middle cerebral artery and right middle cerebral artery respectively. Patients had a burden of medical records with risk factors like hypertension, hyperlipidemia, diabetes or previous mild stroke, with various COVID-19 symptoms (from none symptoms to lethargy). The authors suggest that vascular endothelial dysfunction and coagulopathy are a complication of the ongoing COVID-19 disease. Furthermore, before the world pandemic, the same hospital in the same 2-week period admitted 0.73 patients on average, in comparison to five admitted patients during the pandemic [36]. The large disproportion in the number of patients admitted suggests that neurological manifestations, including ischemic stroke, are very serious complications of the ongoing SARS-CoV-2 virus infection and differential diagnoses should be implemented to hospitals to avoid delays in the diagnosis of concomitant complications. Furthermore, the above-mentioned studies showed that patients with severe infections manifested neurologic symptoms more often.

Merkler et al. published a study in which 1916 COVID-19 patients were enrolled. The study aimed to evaluate the association and risk of acute ischemic stroke in COVID-19 patients in comparison to influenza. Patients were enrolled on two hospitals, with median age 64 years. Patients mostly presented mild symptoms like dyspnea, cough and fever. However, 330 patients had severe infection and required mechanical ventilation. Of 1916 patients, 31 were diagnosed with ischemic stroke (1.6%). Influenza was identified in 1486 patients, in which 1427 had symptoms of viral respiratory illness like: cough, fever and hypoxia. Of 1427 patients, 48 had severe infection that required mechanical ventilation, however, only 3 patients had ischemic stroke (0.2%). The results of studies by Merkler et al. were consistent across multiple analysis and indicated that COVID-19 more likely caused ischemic stroke [37].

Klok et al. published clinical characteristics of 184 patients diagnosed with SARS-CoV-2 infection admitted to the Intensive-Care Unit (ICU), with median age of 64 years. All patients received thromboprophylaxis (nadroparin), with increased doses over time. Among 184 patients, 31% experienced thrombotic complication: pulmonary embolism (25 patients), venous thromboembolic events (3 patients) and ischemic stroke (3 patients) [38].

Lodigiani et al. performed a study, in which the primary outcome of thromboembolic complications, including acute coronary syndrome, venous thromboembolism, ischemic stroke and DIC syndrome due to viral infection was evaluated. Results showed that among 388 patients with confirmed COVID-19, 16 patients had venous thromboembolism, 10 patients were confirmed with pulmonary embolism, 9 patients experienced ischemic stroke and 4 patients were diagnosed with acute coronary syndrome [39].

Yaghi et al. have reported that cryptogenic stroke was confirmed in 32 of 3556 hospitalized patients with positive COVID-19 test. Nearly half of the stroke patients (43.8%) were admitted because of cerebrovascular infarction. D-dimer levels and C-reactive protein were significantly augmented (with median values of 3913 ng/mL and 1011 ng/mL, respectively). The development of stroke with unknown etiology may be related with hypercoagulability caused by SARS-CoV-2 infection [40].

In the study performed by Qin et al. the clinical characteristics and outcomes of COVID-19 patients with and without history of stroke were evaluated. Authors showed that patients with a history of stroke presented more comorbidities, more coagulation disorders and more aggressive inflammatory response. Moreover, those patients had poorer outcomes and higher risk of severe events. Patients with history of stroke had elevated number of neutrophils and interleukin 6 level, which may induce the cytokine storm and augmented, harmful immune system response. However, more severe course of the disease in patients with stroke history may not be associated with viral infection, but with enhanced risk factors and poorer health condition [41].

### 4.2. Molecular Association between SARS-CoV-2 and Ischemic Stroke

Genetically, SARS-CoV-2 shows about 79% similarity with SARS-CoV, and about 50% similarity with the middle-eastern respiratory syndrome coronavirus (MERS-CoV). Studies showed that this new coronavirus enters the human cells by binding to the angiotensin-converting enzyme 2 (ACE2), such as SARS-CoV [42,43]. The parallels between those two viruses are very important in laboratory diagnostics, medical treatment, spreading prevention and clinical characteristics, because since its discovery, the virus has proved itself to be extremely harmful and highly contagious. However, very few studies have yielded any conclusive explanations regarding the virus properties.

ACE2 is mainly expressed in the human airway epithelia, lung parenchyma, kidney cells, heart, testis, vascular endothelial cells, intestinal epithelial cells and brain [44]. Hamming et al. carried out research based on immunohistochemistry testing on 15 different human tissue organs, localized ACE2 in endothelial cells from arteries and veins in all the studied samples, including the brain [45]. These studies, published in 2004 and 2005, demonstrated that SARS-CoV was found in the brain samples of infected patients. Interestingly, virus particles were found mostly in the neurons [46,47,48]. In order to find the means of virus neuroinvasion, Netland et al. performed a study on transgenic mice, infected intranasally with SARS-CoV, and confirmed viral antigen distribution in the brain. Thus suggesting that the virus can enter the brain via the olfactory nerve [49]. There are currently no similar studies that could confirm SARS-CoV-2 brain infection through the olfactory system, however, the similarity between these viruses may suggest that this new coronavirus may invade the brain in the same way.

ACE2 is a part of the renin-angiotensin system (RAS), which is very important in the cardiovascular functions regulation, through the degradation of angiotensin II to angiotensin_1-7_. Experimental studies have shown that angiotensin II induces myocardial hypertrophy, interstitial fibrosis, endothelial dysfunction, hypertension, vasoconstriction, oxidative stress, coagulation and enhances inflammation. The opposite role was shown in the case of angiotensin, which provides anti-inflammatory properties, thus reducing inflammation, fibrosis, migration and infiltration of cells. SARS-CoV-2 binding with the ACE2 receptor leads to its down-regulation, increasing harmful and pathological state development in the host organism [50]. In the CNS, angiotensin II increases blood pressure and releases vasopressin. Moreover, in ACE2 knockout mice models, gene deletion was correlated with the augmented level of superoxides [51].

The severity and mortality of COVID-19 are correlated with the body’s immune response. In a study conducted by Chen et al., most patients diagnosed with COVID-19 had fewer lymphocytes and more c-reactive protein. Furthermore, 52% of enrolled patients had an increased level of serum interleukin 6 (IL6) [52]. Huang et al. conducted a study, in which patients with severe infections, admitted to the intensive care unit, had elevated levels of plasma pro-inflammatory cytokines like IL2, IL10, IL7, granulocyte colony-stimulating factor (GSCF), interferon γ-induced protein (IP10), monocyte chemoattractant protein-1 (MCP1), macrophage inflammatory protein-1-α (MIP1A) and tumor necrosis factor α (TNFα). What is more, the concentration of platelet-derived growth factor (PDGF), vascular endothelial growth factor (VEGF), IL1β, IL8, IL9 and interferon γ (IFN-γ) were elevated in all diagnosed patients [53].

In order to better understand all molecular processes ongoing during viral infection, the molecular mechanisms occurring in various cell types were described, maintaining the events chronology during the infection.

### 4.3. Endothelial Cells

A series of processes causing harmful body response to a viral infection leading to thromboembolic complications, begin with the endothelial cells. ACE2 receptor located in endothelium allows the virus to connect and enter in the cells [44]. Although the adhesion of leukocytes and blood platelets to endothelium is normally prevented, localized pro-inflammatory mediators (cytokines and chemokines), clotting cascade factors, growth factors and nitric oxide effect the reduced barrier integrity [54]. Furthermore, experimental studies showed that TNF and IL1β, which are released from endothelial cells during viral infection, are able to activate endothelial cells via NFκB pathway, which finally induces the new genes expression associated with the inflammatory response, i.e., adhesion molecules like vascular cell adhesion protein 1 (VCAM-1) and intracellular adhesion molecule 1 (ICAM-1) [55]. Furthermore, IL1 and TNF have an ability to increase tissue factor (TF) and plasminogen activator inhibitor, increase the endothelium adhesivity for leukocytes and stimulate the secretion of PDGF. These effects tip the balance between pro- and anti-coagulant properties towards intravascular coagulation [56]. Ongoing inflammation in the endothelium causes changes in vascular permeability and leads to the cells death [54]. Release of DAMPs from injured endothelial cells induces the migration of immune system cells, whose task is to eliminate the pathogen [29].

### 4.4. Leukocytes and Macrophages

A very important role in the viral infection response is played by neutrophils, which are the first cell population that migrates to the damaged area. To eliminate the threat, neutrophils are equipped with various biological features, including chemokines, ROS and proteases (i.e., MPPs). However, all the invasive and aggressive mechanisms responsible for the pathogens elimination also work efficiently with host cells, which can cause damage to the inflamed tissue [29].

Mobilization of macrophages, leukocytes and neutrophils (which constitute an innate immunity system) at the site of infection involves a massive release of cytokines and chemokines in damaged tissue. In the case of the brain tissue, mainly TNFα, IL1β and IL6 were found to be associated with ischemic stroke [48,57]. Cytokines and chemokines, which are released activate endothelium cell adhesion molecules that capture macrophages, leukocytes and neutrophils. The other pro-inflammatory molecule, IFN-γ, may increase the immune response by augmented infiltration of monocytes and lymphocyte into the damaged vessel. Thus elevating the level of surface adhesion molecules and chemokines [58]. Released IL2 possesses an ability to induce T-cell proliferation (which constitute an adaptive immune system) and regulates their development, function and survival, and induces the differentiation of T-helper cells [59]. T-cell development is regulated also by released IL7, which shown the properties to stimulate the recruitment and adhesion of macrophages and monocytes to endothelial cells, and upregulated MCP1 in the endothelium, which is responsible for the antiviral immune response, and the migration and infiltration of monocytes and T-cells [60,61]. Chemokines have a similar effect to MIP1A and IP10 [62,63]. Migration of neutrophils and activation of mast cells are mainly provided by releasing IL8 and IL9, respectively [64,65]. What is more, IL9 may contribute to the augmented production of other pro-inflammatory cytokines in airways, resulting in its hyperresponsiveness [65]. To increase the immune response, GCSF stimulates the generation of the granulocytes, mainly neutrophils, and their release into the bloodstream. However, GCSF has also shown to inhibit the production of TNF and IL8 in monocytes, macrophages and neutrophils, and to induce the expression of IL10, anti-inflammatory cytokine, which enables the reduction of interaction between monocytes and endothelial cells resulting in decreased adhesiveness [66,67]. Strengthening the inflammation as a result of the body’s response to infection is caused by the rapid production of IL6, which is released by microglial, leukocytes, endothelial cells and astrocytes, and is responsible for the stimulation of production of C-reactive protein and fibrinogen. Thus increasing the risk of a thrombotic event. Furthermore, IL6 may accelerate the migration of leukocytes as well as the production of adhesion molecules and chemokines. Studies showed that IL6 is associated with neurovascular dysfunction, neurodegeneration and inflammation of peripheral nerves [28].

As a result of cell death, released DAMPs activate astro- and microglia, thus amplifying the mobilization of immune response cells [29]. The accumulation of immune cells in the vascular wall in response to the viral infection, especially among patients with ischemic risk factors, induces endothelial dysfunction, migration and proliferation of cells, activation of coagulation cascade and production of fibrous plaques. TF, which is activated by cytokines is the key initiator that triggers the coagulation cascade.

### 4.5. Blood Platelets and Coagulation Cascade

Blood platelets are the smallest nucleated blood morphotic elements, which are responsible for the maintaining a hemostasis process. In addition to that, platelets are the only cytoplasmic fragments of megakaryocytes, they are equipped with large number of receptors and biologically active compounds that interact with vascular microenvironment. Under physiological conditions, platelets freely circulate in bloodstream without interacting with endothelium. This property is ensured by a glycoproteins layer and proteoglycans present between endothelium and blood, known as the glycocalyx [68]. However, inflammatory mediators released during viral infection, such as TNFα and lipopolysaccharide (LPS), can cause degradation of the glycocalyx, thus regulating the permeability of endothelium.

The injured endothelium expose TF, which triggers the coagulation cascade. Firstly, TF binds with serine protease factor VIIa, which further activates factor X and factor IX, resulting in thrombin generation in the final [69]. The positive thrombin feedback brings a blood platelet activation. Activated platelets change their shape in order to expose their adhesion receptors and to release granular content, pro-inflammatory cytokines and chemokines, and other activators (i.e., ADP, vWF, thromboxane A2) that enhance thrombus formation [68]. Simultaneously, during the progression of the coagulation cascade, factor XIIa cleaves plasma prekallikrein to form the active serine protease plasma kallikrein that generates bradykinin. Its binding to endothelium resulting in the induction of glial activation, enhancing an inflammation and neuronal death, which in turn enhances the secretion of DAMPs [70].

**Figure 2:**
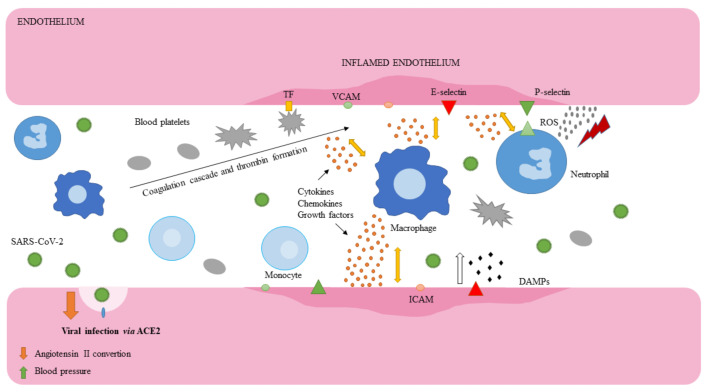
Interaction of endothelium, immune and hemostatic cells during viral infection.

Activated blood platelets interact with leukocytes via glycoprotein P-selectin platelets and its ligand (P-selectin glycoprotein ligand-1; PSGL-1) on leukocytes, and support their migration to inflamed endothelium [68]. Adhesive molecules on endothelium (E-selectin, P-selectin, VCAM-1) trap the rolling leukocytes [71]. During the accumulation of immune and hemostatic cells, thrombin generates the insoluble fibrin from fibrinogen [69]. Furthermore, this effect is enhanced by cytokines that stimulate the plasminogen activator inhibitor-1 (PAI-1), that reduces the fibrinolysis efficiency and effectivity [56]. The ongoing recruitment of platelets and successive infiltration of leukocytes, neutrophils and macrophages cause thickening of plaque that blocks the blood flow, resulting in the ischemic event (Figure 2) [21,70].

Severe acute respiratory syndrome coronavirus 2 (SARS-CoV-2) infects human cells via the angiotensin-converting enzyme 2 (ACE2) receptor. Neutrophils migrate to the infected area in order to eliminate the pathogen. Release of biologically active compounds (i.e., chemokines, reactive oxygen species—ROS) stimulates the inflammation, causing mobilization of other immune system cells. Dead cells from the injured zone release damaged-associated molecular patterns (DAMPs), which activate microglia, inducing the migration of macrophages, leukocytes and neutrophils. The ongoing endothelial dysfunction activates the coagulation cascade via tissue factor (TF). Generated thrombin stimulates blood platelets activation and shape change. Thus exposing adhesion receptors and secreting granular content, enhancing inflammation and coagulation. Activated platelets interact with leukocytes and support their migration to the damaged area. The interaction among endothelium, immune system and hemostatic cells enhances the ischemia and inflammation, simultaneously reducing the fibrinolysis effectivity and efficiency.

## 5. Pharmacological Treatment

Pharmacological treatment of stroke patients must be matched with the stroke type. That is why a detailed medical interview and examination have to be performed before drug supplementation. Ischemic stroke patients mostly received thrombolytic therapy. Clinical trials showed that recombinant tissue plasminogen activator (rt-PA, also named alteplase) administered in maximal 4.5 h after onset of symptoms, significantly reduce hypoxia and improve patients outcomes [72]. According to the Early Management of Patients With Ischemic Stroke guidelines, the combination of rt-PA with antiplatelet medicaments (excluding heparin, thrombin inhibitors, factor Xa inhibitors and GPIIb/IIIa inhibitors) is recommended because of their beneficial character [73]. Due to the National Institutes of Health guidelines, hospitalized adult patients should be administered with venous thromboembolic events (VTE) prophylaxis, if hematologic and coagulation parameters indicate the possibility of thrombotic complications, or patients are at high risk of thromboembolic event. What is more, patients receiving antiplatelet and anticoagulant therapies before COVID-19 diagnosis should continue the treatment. However, available data are insufficient to recommend the use of thrombolytics and anticoagulant drugs.

In the case of SARS-CoV-2 infection, there is no antiviral agent for COVID-19, however, several medicaments, including Remdesivir, Chloroquine, Lopinavir, Rotinavir and other HIV Protease Inhibitors, are evaluated as a potential antiviral drug (Table 1). Administration and selection of anticoagulant or antiplatelet drug for COVID-19 patients should be always considered to potential drug–drug interactions. For this reason, The University of Liverpool collated a list of drug interactions for medical personnel [74].

## 6. Conclusions

Numerous studies showed that COVID-19 may cause thromboembolic complications, which lead to many vascular disorders, including ischemic stroke. The rapidly growing number of case-reports demonstrates the need for more detailed medical examination of patients, especially those with severe infections. Oxygen and nutrient shortages caused by a viral infection, along with the release of cytokines and chemokines, migration and influx of immune defense cells, their interactions with endothelium and accumulation in the damaged area, activation of the coagulation system and generation of thrombus result in many thromboembolic complications.

## Figures and Tables

**Table 1:** Potential antiviral drugs under evaluation for the treatment of coronavirus disease 2019 (COVID-19) [74].

Remdesivir	Intravenous prodrug responsible for inhibiting viral replication via binding to the viral RNA polymerase.
Chloroquine/Hydroxychloroquine	Antimalarial drug, which inhibits the fusion of virus with host cell membranes. In vitro studies showed that both drugs may block the viral transport from endosomes to endolysosomes, thus regulating the releasement of viral genome. Chloroquine has an ability to inhibits glycosylation of ACE2 receptor, thus interfering the viral linkage.
Lopinavir/Ritonavir	Lopinavir/Ritonavir inhibits the activity of proteases responsible for replication of SARS-CoV-2.
